# PKM2: A Potential Regulator of Rheumatoid Arthritis via Glycolytic and Non-Glycolytic Pathways

**DOI:** 10.3389/fimmu.2019.02919

**Published:** 2019-12-18

**Authors:** Danyi Xu, Junyu Liang, Jin Lin, Chaohui Yu

**Affiliations:** ^1^Department of Rheumatology, The First Affiliated Hospital, College of Medicine, Zhejiang University, Hangzhou, China; ^2^Department of Gastroenterology, The First Affiliated Hospital, College of Medicine, Zhejiang University, Hangzhou, China

**Keywords:** rheumatoid arthritis, PKM2, glycolysis, protein kinase, tumor

## Abstract

Immunometabolism provides a new perspective on the pathogenesis of rheumatoid arthritis (RA). In recent years, there have been investigations focusing on the role of intracellular glucose metabolism in the pathogenesis of RA. Previous studies have shown that glycolysis of synovial tissue is increased in RA patients, while glycolysis inhibitors can significantly inhibit synovitis. Pyruvate kinase (PK) is a key enzyme in glycolysis, catalyzing the final rate-limiting step in the process. An isoform of PK, PKM2, provides favorable conditions for the survival of tumor cells via its glycolytic or non-glycolytic functions and has become a potential therapeutic target in tumors. RA synovium has the characteristic of tumor-like growth, and, moreover, increased expression of PKM2 was identified in the synovial tissue of RA patients in recent studies, indicating the underlying role of PKM2 in RA. PKM2 has potential value as a new therapeutic target or biomarker for RA, but its exact role in RA remains unclear. In this review, the properties of PKM2 and existing research concerning PKM2 and RA are thoroughly reviewed and summarized, and the possible role and mechanism of PKM2 in RA are discussed.

## Introduction

Rheumatoid arthritis (RA) is a common autoimmune disease with the pathological characteristics of invasive synovitis, pannus formation, and articular cartilage destruction. An imbalance between proliferation and apoptosis plays an important role in the pathogenesis of RA, and this tumor-like characteristic of the rheumatoid synovium has become a hot issue in RA research in recent years (1). However, its detailed pathogenesis remains unclear.

Currently, investigations on RA mainly focus on immunology, genetics, and cell biology, whereas studies on the pathogenesis of RA from the perspective of glucose metabolism have not been widely considered. However, glucose metabolism plays a pivotal role in the pathogenesis of RA. Previous studies found that the synovial tissues of RA patients were hypoxic and that this was accompanied by an increase in glycolytic enzyme gene expression and glycolytic activity (2). The key enzymes of glycolysis, such as glucose phosphate isomerase (GPI) (3), aldolase (ALD) (4), and triose phosphate isomerase (TPI) (5), can take part in autoimmune reaction in RA as antigens.

Pyruvate kinase (PK) is a key rate-limiting enzyme of glycolysis that irreversibly catalyzes the conversion of phosphoenolpyruvate (PEP) to pyruvate (6). PKM2, an isoform of PK, is highly expressed in tumor cells, leading to increased glucose uptake, a transition from oxidative phosphorylation to glycolysis, and accumulation of glucose metabolites. It thus provides favorable and necessary conditions for the growth and survival of tumor cells (7). Alterations in energy metabolism are one of the most important differences between tumor cells and normal cells (8). PKM2 is a key factor leading to this effect, and it has become a potential target for the treatment of tumors. Synovial tissue in RA has been characterized as tumor-like proliferation. In recent years, a few studies have confirmed that the expression level of PKM2 in the synovial tissue of RA is significantly higher than that in patients with osteoarthritis (OA) (9), suggesting the potential role of PKM2 in RA, though further studies are still lacking. However, no summary has been made to help enhance our understanding in this area. Here, the characteristics of PKM2 and the existing evidence on PKM2 and RA are reviewed, and the potential role and mechanism of PKM2 in RA are discussed.

## Biological Characteristics of PKM2

PK catalyzes the final rate-limiting and irreversible step in glycolysis, which produces pyruvate and ATP. In mammals, there are four PK isoforms: PKL, PKR, PKM1, and PKM2, expressing in different cells and tissues (10). PKM1 and PKM2 are encoded by the PKM gene and are generated through alternative splicing of two exons in PKM pre-mRNA (11). PKM1 is expressed in most well-differentiated tissues, whereas PKM2 is expressed in proliferative cells, such as embryonic cells, adult stem cells, and cancer cells, especially.

PKM2 can take dimer or tetramer forms with different functions, and these can be converted into each other (12). The tetramer form has a high affinity with its metabolic substrate PEP, while the dimer form has a low affinity with PEP, which means that the tetramer form has a high glycolytic enzyme activity under physiological conditions, while the dimer form has low enzyme activity (12). The tetramer form of PKM2 can efficiently promote glycolysis and energy production (13), while the presence of the low-activity PKM2 dimer stops the conversion from PEP to pyruvate, which leads to the accumulation of glucose metabolites in the upstream and the accumulation of a large number of precursor substances for the synthesis of macromolecules (7).

## Glycolytic Function of PKM2

It was found that although there is only one exon difference between the structures of PKM1 and PKM2, they have significant differences in function. Under physiological conditions, PKM1 constitutively exists in the form of a highly active tetramer, while PKM2 can exist in the dimer and tetramer forms (14). Small molecules and metabolites, such as fructose-1,6-bisphosphate (FBP) (15), Succinyl-5-aminoimidazole-4-carboxamide-1-ribose-50-phosphate (SAICAR) (16), serine (17), etc., can promote the tetramerization of PKM2. Meanwhile, PKM2 activity can be inhibited by posttranslational modification, such as phosphorylation (18), acetylation (19), and oxidation (20). The enzymatic activity of PKM2 can be regulated by endogenous mechanisms, so that proliferating cells may choose for PKM2 to allow PK enzyme activity to be turned on or off as the environment changes, thus providing metabolic flexibility.

**Figure 1:**
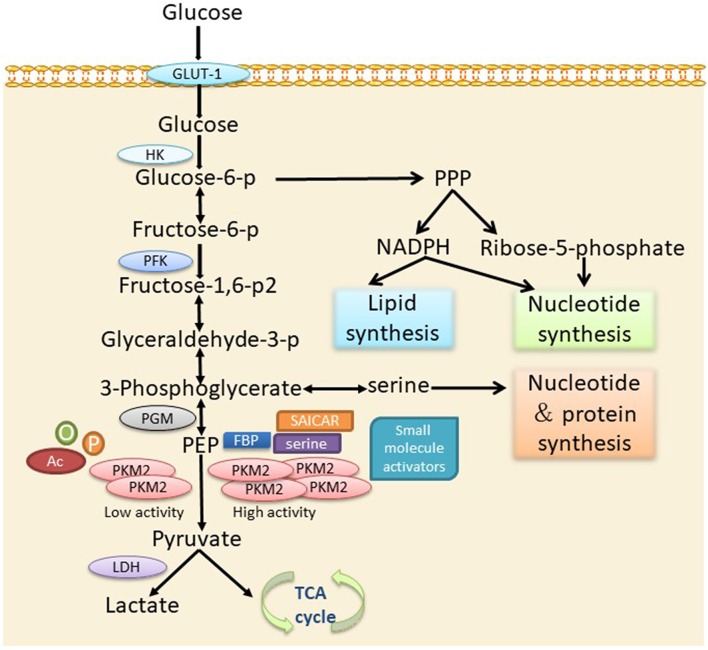
The role of PKM2 in cellular metabolism via the glycolytic pathway. PKM2 is converted into an active tetramer under activation by serine, FBP, SAICAR, or small molecules, which promotes the conversion of PEP into pyruvate. Pyruvate enters the TCA cycle of the mitochondria and produces ATP through oxidative phosphorylation. In the absence of allosteric activators or post-transcriptional modifications, PKM2 presents mainly in an inactive dimer form, leading to the accumulation of glycolytic intermediates to meet the needs of the biosynthetic precursors of activated or proliferating cells. p, phosphorylation; Ac, acetylation; o, oxidation.

The significant alteration in the energy metabolism of tumor cells is characterized by the increase of glucose consumption and the conversion of a large amount of glucose into lactate through glycolysis under aerobic conditions, namely aerobic glycolysis, also known as the Warburg effect (21). Another important feature of tumor cell metabolism is the rapid synthesis of large amounts of lipids, proteins, and nucleotides (8), which are necessary for the rapid growth of tumor cells. PKM2 is the major factor that leads to the change in tumor cell metabolism. The presence of the dimer and tetramer forms of PKM2 and their mutual conversion meets the large energy and anabolic substrate supply demands of tumor cells. Previous findings have confirmed that a dimer form PKM2 is commonly expressed in tumors (22). When tumor cells are short of energy, PKM2 transforms from a dimer structure to a tetramer structure and provides enough energy for the cells. When energy is sufficient, it can intercept the conversion of glucose to lactate in the form of a dimer, thus providing sufficient substrates for the anabolism of tumor cells. Therefore, PKM2 can optimize the supply of energy and synthetic substrates for tumor cells, contributing to tumor cell survival and proliferation in a complex microenvironment (23) (Figure 1).

## Non-Glycolytic Function of PKM2

Recent studies have shown that PKM2 has not only a glycolytic function but also has non-glycolytic function. Under various conditions, PKM2 can be depolymerized from a tetramer to a dimer and translocated to the nucleus, mitochondrial membrane, or outside the cell. PKM2 acts as a protein kinase by phosphorylating various protein substrates at both serine/threonine and tyrosine residues. PKM2 can use its metabolic substrate, PEP, as a phosphate donor for phosphorylation of a variety of target proteins, including STAT3, histone H3, myosin light chain 2 (MLC2), Bub3, and ERK1/2 (24). The translocated PKM2 dimer is involved in the regulation of gene transcription, metabolic reprogramming, mitosis, apoptosis, and other important life events by transcriptional activation, modulating signal transduction, or regulation of the phosphorylation of important proteins, endowing tumor cells with growth advantages (24).

Although protein kinase substrates of PKM2 are being continuously identified, PKM2-mediated gene expression has been challenged by other studies. A lack of a PKM2 gene in mouse models did not inhibit tumor growth (25), and furthermore, PKM2 acting as a PEP-dependent protein kinase was not proved in a recent study (26). Therefore, PKM2 may be directly or indirectly involved in the transcriptional activation, signal transduction, or phosphorylation of some proteins. The specific mechanism demands further exploration.

## Existing Evidence Concerning PKM2 and RA

Activated synovial cells have the biological behavior characteristics of excessive proliferation, migration, and invasion and form tumor-like pannus, which forms a low-oxygen microenvironment (27, 28). Hypoxia alters cellular bioenergetics by promoting a switch to glycolysis so as to efficiently produce enough ATP to support enhanced synovial proliferation and pannus formation. Compared to OA-fibroblast-like synoviocytes (FLS), the glycometabolism shifted toward glycolysis in RA-FLS (29). The increased fluorodeoxyglucose (FDG) uptake in swollen joints in patients with RA reported in several studies represents the up-regulation of glycolysis (30, 31). Further studies have confirmed that fibroblasts and activated macrophages contribute to the high level of FDG accumulation in the pannus (31). The finding of a marked increase in both glyceraldehyde-3-phosphate dehydrogenase (GAPD) and LDH activities in rheumatoid synoviocytes also suggests the up-regulation of glycolysis (32).

So far, our knowledge on PKM2 and RA is limited; however, several studies have demonstrated that PKM2 may be involved in the pathogenesis of RA. As a key enzyme in the glycolytic pathway, the PKM2 expression of RA-FLS was significantly increased under hypoxic conditions, while inhibition of glycolytic activity by glycolytic inhibitor 3-(3-pyridinyl)-1-(4-pyridinyl)-2-propen-1-one(3PO) dramatically reverse pro-inflammatory mechanisms including invasion, migration, cytokine secretion, and the signaling pathways of hypoxia-inducible factor-1α(HIF-1α), pSTAT3, and Notch-1IC (33). Another study confirmed that PKM2 is more highly expressed in the lining layer, sublining layer, and vasculature of RA synovial tissue compared to OA (9). TLR2-activation can induce an increased glycolysis:respiration ratio in RA-FLS and enhanced PKM2 nuclear translocation. Similarly, 3PO inhibits TLR2-induced inflammation and signaling pathways (9). In a proteomic analysis of RA-FLS, a total of 1,633 and 1,603 protein spots were examined in RA patients and controls, respectively. Among them, 33 proteins were over-expressed by more than 3-fold, and 3 proteins, namely α-enolase, GRP75, and PKM2, were verified by Western blot (34). Therefore, PKM2 may be involved in the occurrence and development of RA, and further explorations to clarify its exact role and mechanism in RA will be of great significance.

## Potential Role and Mechanism of PKM2 in RA

**Figure 2:**
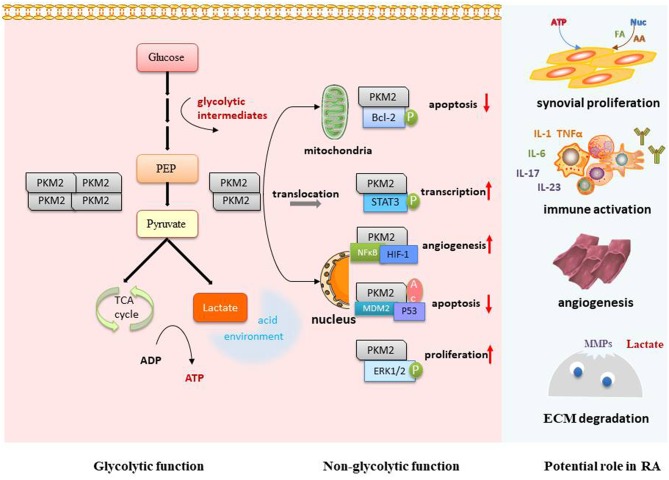
Potential role and mechanism of PKM2 in RA. PKM2 can optimize the supply of energy and synthetic substrates for proliferative synoviocytes and activated immune cells via its glycolytic regulation function. The dimer form of PKM2 can interact with important RA transcription factors, such as STAT3, Bcl-2, HIF-1, and Erk1/2, so as to further regulate cell proliferation, apoptosis, angiogenesis, and immune activation. The local acidic microenvironment caused by increased glycolysis is favorable for synoviocyte invasion, MMP-1 activation, and angiogenesis. p, phosphorylation; Ac, acetylation; FA, fatty acid; AA, amino acid; Nuc, nucleotide; ECM, extracellular matrix.

PKM2 has become a hotspot in the field of tumor research. However, there have been few studies on PKM2 and RA, and its function and mechanism in the development of RA remain unclear. Based on the tumor-like invasion characteristics of synovial tissue, we refer to studies on tumor and PKM2 and try to discuss the possible mechanism in terms of the following aspects so as to clarify the areas worthy of research interest in the future (Figure 2).

### Glycolytic Regulation in RA

Increased glycolysis provides sufficient energy to maintain rapid cell proliferation for synovial tissue in RA (29). On the other hand, many intermediate metabolic products produced during glycolysis are necessary substrates for the synthesis of cellular macromolecules for synoviocyte proliferation. PKM2 may thereby optimize the supply of energy and synthetic substrates for synoviocytes to maintain the tumor-like proliferation.

The local acid microenvironment caused by increased glycolytic activity (32) can further aggravate the pathological process of RA. The acid environment is favorable for synoviocyte survival, as it is for tumor cells. It was found that lactate, as the end product of glycolysis, could trigger invasiveness of FLS (33). The large amount of lactate leaves tissues in an acid environment, capable of destroying the extracellular matrix either directly or by activating metalloproteinases (35). Lactate and pyruvic acid can stimulate the expression of vascular endothelial growth factor (VEGF) and HIF-1α, which are critical for angiogenesis (36). On the other hand, acidosis itself can cause mutations and aberrations that prevent DNA repair, as well as mutations in normal cells (37). Therefore, PKM2 may regulate glycolysis to make synovial tissues in an acid environment that is conducive to their proliferation and invasion.

The inflammatory response is an energy-intensive process that requires a transition from resting to a highly active metabolic state. Under inflammatory conditions, metabolic reprogramming is guided by the efficient generation of ATP and the synthesis of macromolecules for the activation and proliferation of immune cells. As with tumor cells, highly active immune cells also show metabolic changes from oxidative phosphorylation to glycolysis. Immune cell metabolism regulation has become an attractive potential therapeutic target for inflammatory and immune diseases. Studies have shown that glycolysis is associated with T cell differentiation (38, 39) and the production of IFNγ-1 (40). In RA synovial tissues and plasma, disorders of some glycolytic enzymes can induce the activation of immune cells (41, 42). Recent studies have shown that PKM2 is significantly increased in LPS-activated macrophages (43) and participates in regulating macrophage polarization (44). Hence, PKM2 may be a potential modulator of immune cell metabolism and function; this still needs to be further clarified.

### Potential Protein Kinase Targets

It has been found that PKM2 can interact with a variety of proteins as a protein kinase. However, so far, no investigators have conducted relevant studies on PKM2 as a protein kinase in RA. Therefore, by referring to the findings of PKM2 in tumor studies, we discuss several possible targets of PKM2 as a protein kinase in RA.

#### STAT3

STAT is a family of cytoplasmic proteins that can trigger the transcription of corresponding target genes (45). It was found that PKM2 could phosphorylate STAT3 at Tyr705 and promote MEK5 transcriptional activity (46). Inhibition of PKM2 expression could effectively attenuate the neuropathic pain and inflammatory responses induced by CCI in rats, possibly by regulating ERK and STAT3 signaling pathways (47). LPS promoted PKM2 binding to the STAT3 promoter to enhance STAT3 expression and its subsequent nuclear translocation, inducing TNF-α and IL-1β production and cell proliferation in CRC cells (48). Targeting the JAK/STAT signaling pathway is a new way of treating RA, and further studies will be needed to confirm whether inhibiting PKM2 in RA can reduce cell proliferation and inflammatory cytokine secretion via the STAT pathway.

#### Bcl-2

Bcl-2 is a product of the B-lymphoma-2 gene, that regulates cell survival and inhibits apoptosis (49). Under oxidative stress, PKM2 translocates to the mitochondrial outer membrane, where heat shock protein (HSP)90α1 mediates conformational changes in PKM2 then interacts with and phosphorylates Bcl-2 at thr69. This phosphorylation prevents the binding of BTB–CUL3–RBX1 (BCR) E3 ligase to Bcl-2, thereby inhibiting the degradation of Bcl-2 and inhibiting the apoptosis of tumor cells (50). Knockdown of PKM2 induced apoptosis and autophagy in A549 cells, and this was dependent on decreased expression of Bcl-2 (51). The IL-17/STAT3 pathway may promote the survival and proliferation of FLSs via upregulating the expression of Bcl-2 in RA (52). The overexpression of Bcl-2 may contribute to the reduced apoptosis of synoviocytes and peripheral B cells in RA patients (53). The relations between increased expression of Bcl-2 and PKM2 in synovial tissues deserve more investigation to clarify them further.

#### HIF-1α

HIF-1α is a major regulator of the transition from oxidative phosphorylation to anaerobic glycolysis (54). PKM2 was able to interact with NF-κB and HIF-1α in the nucleus and activate the expression of the target gene VEGF-A, thereby promoting tumor angiogenesis (55). Meanwhile, in macrophages, LPS-induced PKM2 could enter into a complex with HIF-1α, which directly bound to the IL-1β promoter, inhibiting LPS-induced HIF-1α and IL-1β, as well as the expression of a range of other HIF-1α-dependent genes (43). HIF-1α is highly expressed in the synovium of RA patients. HIF-1α can promote the migration of inflammatory cells to the RA synovium, and the expression of matrix metalloproteinase (MMP)-1 is up-regulated by HIF-1α under hypoxia (56). The “hypoxia-HIF-1α-VEGF” signaling pathway plays an important role in the formation of new blood vessels in RA, so targeting HIF-1α via PKM2 could make a lot of sense.

#### p53

p53 can work as a hub among a variety of intracellular signal transduction pathways, inhibiting cell proliferation, terminating cell processes, and inducing apoptosis (57). Recent studies have found that PKM2 may be involved in the regulation of p53 in tumor cells. In MCF7 cells exposed to DNA-damaging agent, PKM2 inhibited transactivation of the p21 gene by preventing p53 binding to the p21 promoter, leading to a nonstop G1 phase (58). Dimeric PKM2 has been found to bind directly with both p53 and MDM2 and to promote MDM2-mediated p53 ubiquitination (59). PKM2 gene silencing suppresses proliferation and promotes apoptosis in LS-147T and SW620 cells, accompanied by increased p53 and p21 expression (60). Most studies suggest that p53 expression is decreased in RA-FLS and synovial tissue (61). Collagen-induced arthritis (CIA) was more severe and with more joint destruction in p53^−/−^ mice than in wild type mice due to a reduction in synovial cell apoptosis (62). The future task is to clarify the regulation effect and molecular mechanism of PKM2 on p53 in synoviocytes.

#### ERK1/2

An ERK1/2 signaling pathway is an important intracellular pro-proliferation and anti-apoptosis pathway (63). In cancer cells, a PKM2-SAICAR complex phosphorylated and activated ERK1/2, which in turn sensitized PKM2 for SAICAR binding through phosphorylation (64). Additionally, PKM2-SAICAR was necessary to induce sustained ERK1/2 activation and mitogen-induced cell proliferation. In Panc-1 and Sw1990 pancreatic cancer cells, the expression levels of the p-ERK1/2 and p-p38 of the MAPK pathway in the PKM2 siRNA groups were markedly down-regulated (65). ERK1/2 is widely distributed in the synovial tissues of RA and is involved in the signal transduction process of synoviocytes (66). Clarifying the effect of PKM2 on ERK1/2 is helpful for exploring its role in the pathogenesis of RA.

## Conclusion

In summary, glucose metabolism, especially increased glycolysis, plays a pivotal role in the pathogenesis of RA. Recent studies have confirmed the overexpression of PKM2 in RA synovium, suggesting a potential role for PKM2 in RA, though the exact role and mechanism have not been systematically studied. We infer that PKM2 may participate in RA pathogenesis through glycolytic or non-glycolytic pathways and that more investigations to are needed to clarify this further. PKM2 may be a potential therapeutic target due to its regulation of the immunometabolism and key signaling proteins in RA, and a small-molecule drug targeting the regulation of PKM2 activity or protein expression may be a novel approach for RA treatment.

## Author Contributions

DX and JLia wrote the review. CY and JLin edited the manuscript. All authors have read and approved the final manuscript.

### Conflict of Interest

The authors declare that the research was conducted in the absence of any commercial or financial relationships that could be construed as a potential conflict of interest.

## Abbreviations

ALD: aldolase
BCR: BTB–CUL3–RBX1
FBP: fructose-1,6-bisphosphate
FLS: fibroblast-like synoviocytes
FDG: fluorodeoxyglucose
GPI: glucose phosphate isomerase
GAPD: glyceraldehyde-3-phosphate dehydrogenase
GLUT-1: glucose transporter protein-1
HSP: heat shock protein
HIF-lα: hypoxia-inducible factor-1α
HK: hexokinase
LDH: lactic dehydrogenase
MLC2: myosin light chain 2
MMP: matrix metalloproteinase
NADPH: nicotinamide adenine dinucleotide phosphate
OA: osteoarthritis
PK: pyruvate kinase
PEP: phosphoenolpyruvate
PFK: phosphofructokinase
PGM: phosphoglycerate mutase
PPP: pentose phosphate pathway
RA: rheumatoid arthritis
SAICAR: succinyl-5-aminoimidazole-4-carboxamide-l-ribose-50-phosphate
TPI: triose phosphate isomerase
TCA: tricarboxylic acid
VEGF: vascular endothelial growth factor
3PO: 3-(3-pyridiny1)-1-(4-pyridinyl)-2-propen-1-one.

## References

[1] You S Koh JH Leng L Kim WU Bucala R. The tumor-like phenotype of rheumatoid synovium: molecular profiling and prospects for precision medicine. Arthritis Rheumatol. (2018) 70:637–52. 10.1002/art.40406 29287304 PMC5920713

[2] Falconer J Murphy AN Young SP Clark AR Tiziani S Guma M. Review: synovial cell metabolism and chronic inflammation in rheumatoid arthritis. Arthritis Rheumat. (2018) 70:984–99. 10.1002/art.40504 29579371 PMC6019623

[3] van Gaalen FA Toes RE Ditzel HJ Schaller M Breedveld FC Verweij CL. Association of autoantibodies to glucose-6-phosphate isomerase with extraarticular complications in rheumatoid arthritis. Arthritis Rheum. (2004) 50:395–9. 10.1002/art.20028 14872481

[4] Ukaji F Kitajima I Kubo T Shimizu C Nakajima T Maruyama I. Serum samples of patients with rheumatoid arthritis contain a specific autoantibody to “denatured” aldolase A in the osteoblast-like cell line, MG-63. Ann Rheum Dis. (1999) 58:169–74. 10.1136/ard.58.3.169 10364915 PMC1752850

[5] Xiang Y Sekine T Nakamura H Imajoh-Ohmi S Fukuda H Nishioka K. Proteomic surveillance of autoimmunity in osteoarthritis: identification of triosephosphate isomerase as an autoantigen in patients with osteoarthritis. Arthritis Rheum. (2004) 50:1511–21. 10.1002/art.20189 15146421

[6] Barnett JA. A history of research on yeasts 5: the fermentation pathway. Yeast. (2003) 20:509–43. 10.1002/yea.986 12722184

[7] Christofk HR Vander Heiden MG Harris MH Ramanathan A Gerszten RE Wei R. The M2 splice isoform of pyruvate kinase is important for cancer metabolism and tumour growth. Nature. (2008) 452:230–3. 10.1038/nature06734 18337823

[8] Hanahan D Weinberg RA. Hallmarks of cancer: the next generation. Cell. (2011) 144:646–74. 10.1016/j.cell.2011.02.013 21376230

[9] McGarry T Biniecka M Gao W Cluxton D Canavan M Wade S. Resolution of TLR2-induced inflammation through manipulation of metabolic pathways in Rheumatoid Arthritis. Sci Rep. (2017) 7:43165. 10.1038/srep43165 28225071 PMC5320554

[10] Takenaka M Yamada K Lu T Kang R Tanaka T Noguchi T. Alternative splicing of the pyruvate kinase M gene in a minigene system. Eur J Biochem. (1996) 235:366–71. 10.1111/j.1432-1033.1996.00366.x 8631356

[11] Noguchi T Inoue H Tanaka T. The M1- and M2-type isozymes of rat pyruvate kinase are produced from the same gene by alternative RNA splicing. J Biol Chem. (1986) 261:13807–12. 3020052

[12] Ikeda Y Noguchi T. Allosteric regulation of pyruvate kinase M2 isozyme involves a cysteine residue in the intersubunit contact. J Biol Chem. (1998) 273:12227–33. 10.1074/jbc.273.20.12227 9575171

[13] Mazurek S Zwerschke W Jansen-Durr P Eigenbrodt E. Effects of the human papilloma virus HPV-16 E7 oncoprotein on glycolysis and glutaminolysis: role of pyruvate kinase type M2 and the glycolytic-enzyme complex. Biochem J. (2001) 356(Pt 1):247–56. 10.1042/bj3560247 11336658 PMC1221834

[14] Dombrauckas JD Santarsiero BD Mesecar AD. Structural basis for tumor pyruvate kinase M2 allosteric regulation and catalysis. Biochemistry. (2005) 44:9417–29. 10.1021/bi0474923 15996096

[15] Christofk HR Vander Heiden MG Wu N Asara JM Cantley LC. Pyruvate kinase M2 is a phosphotyrosine-binding protein. Nature. (2008) 452:181–6. 10.1038/nature06667 18337815

[16] Keller KE Tan IS Lee YS. SAICAR stimulates pyruvate kinase isoform M2 and promotes cancer cell survival in glucose-limited conditions. Science. (2012) 338:1069–72. 10.1126/science.1224409 23086999 PMC3527123

[17] Morgan HP O'Reilly FJ Wear MA O'Neill JR Fothergill-Gilmore LA Hupp T. M2 pyruvate kinase provides a mechanism for nutrient sensing and regulation of cell proliferation. Proc Natl Acad Sci USA. (2013) 110:5881–6. 10.1073/pnas.1217157110 23530218 PMC3625322

[18] Hitosugi T Kang S Vander Heiden MG Chung TW Elf S Lythgoe K. Tyrosine phosphorylation inhibits PKM2 to promote the Warburg effect and tumor growth. Sci Signal. (2009) 2:ra73. 10.1126/scisignal.2000431 19920251 PMC2812789

[19] Lv L Li D Zhao D Lin R Chu Y Zhang H. Acetylation targets the M2 isoform of pyruvate kinase for degradation through chaperone-mediated autophagy and promotes tumor growth. Mol Cell. (2011) 42:719–30. 10.1016/j.molcel.2011.04.025 21700219 PMC4879880

[20] Anastasiou D Poulogiannis G Asara JM Boxer MB Jiang JK Shen M. Inhibition of pyruvate kinase M2 by reactive oxygen species contributes to cellular antioxidant responses. Science. (2011) 334:1278–83. 10.1126/science.1211485 22052977 PMC3471535

[21] Warburg O. On the origin of cancer cells. Science. (1956) 123:309–14. 10.1126/science.123.3191.309 13298683

[22] Altenberg B Greulich KO. Genes of glycolysis are ubiquitously overexpressed in 24 cancer classes. Genomics. (2004) 84:1014–20. 10.1016/j.ygeno.2004.08.010 15533718

[23] Wong N Ojo D Yan J Tang D. PKM2 contributes to cancer metabolism. Cancer Lett. (2015) 356(2 Pt A):184–91. 10.1016/j.canlet.2014.01.031 24508027

[24] Lu Z Hunter T. Metabolic kinases moonlighting as protein kinases. Trends Biochem Sci. (2018) 43:301–10. 10.1016/j.tibs.2018.01.006 29463470 PMC5879014

[25] Dayton TL Gocheva V Miller KM Israelsen WJ Bhutkar A Clish CB. Germline loss of PKM2 promotes metabolic distress and hepatocellular carcinoma. Genes Dev. (2016) 30:1020–33. 10.1101/gad.278549.116 27125672 PMC4863734

[26] Hosios AM Fiske BP Gui DY Vander Heiden MG. Lack of evidence for PKM2 protein kinase activity. Mol Cell. (2015) 59:850–7. 10.1016/j.molcel.2015.07.013 26300261 PMC4548833

[27] Ng CT Biniecka M Kennedy A McCormick J Fitzgerald O Bresnihan B. Synovial tissue hypoxia and inflammation *in vivo*. Ann Rheum Dis. (2010) 69:1389–95. 10.1136/ard.2009.119776 20439288 PMC2946116

[28] Hitchon CA El-Gabalawy HS Bezabeh T. Characterization of synovial tissue from arthritis patients: a proton magnetic resonance spectroscopic investigation. Rheumatol Int. (2009) 29:1205–11. 10.1007/s00296-009-0865-z 19184029

[29] Garcia-Carbonell R Divakaruni AS Lodi A Vicente-Suarez I Saha A Cheroutre H. Critical role of glucose metabolism in rheumatoid arthritis fibroblast-like synoviocytes. Arthritis Rheumatol. (2016) 68:1614–26. 10.1002/art.39608 26815411 PMC4963240

[30] Kubota K Ito K Morooka M Mitsumoto T Kurihara K Yamashita H. Whole-body FDG-PET/CT on rheumatoid arthritis of large joints. Ann Nucl Med. (2009) 23:783–91. 10.1007/s12149-009-0305-x 19834653

[31] Matsui T Nakata N Nagai S Nakatani A Takahashi M Momose T. Inflammatory cytokines and hypoxia contribute to 18F-FDG uptake by cells involved in pannus formation in rheumatoid arthritis. J Nucl Med. (2009) 50:920–6. 10.2967/jnumed.108.060103 19443596

[32] Young SP Kapoor SR Viant MR Byrne JJ Filer A Buckley CD. The impact of inflammation on metabolomic profiles in patients with arthritis. Arthritis Rheum. (2013) 65:2015–23. 10.1002/art.38021 23740368 PMC3840700

[33] Biniecka M Canavan M McGarry T Gao W McCormick J Cregan S. Dysregulated bioenergetics: a key regulator of joint inflammation. Ann Rheum Dis. (2016) 75:2192–200. 10.1136/annrheumdis-2015-208476 27013493 PMC5136702

[34] Li XJ Xu M Zhao XQ Zhao JN Chen FF Yu W. Proteomic analysis of synovial fibroblast-like synoviocytes from rheumatoid arthritis. Clin Exp Rheumatol. (2013) 31:552–8. 23739258

[35] Gillies RJ Gatenby RA. Adaptive landscapes and emergent phenotypes: why do cancers have high glycolysis? J Bioenerget Biomembr. (2007) 39:251–7. 10.1007/s10863-007-9085-y 17624581

[36] Walenta S Mueller-Klieser WF. Lactate: mirror and motor of tumor malignancy. Semin Radiat Oncol. (2004) 14:267–74. 10.1016/j.semradonc.2004.04.004 15254870

[37] Gatenby RA Gillies RJ. Why do cancers have high aerobic glycolysis? Nat Rev Cancer. (2004) 4:891–9. 10.1038/nrc1478 15516961

[38] Chang CH Curtis JD Maggi LB Jr Faubert B Villarino AV O'Sullivan D. Posttranscriptional control of T cell effector function by aerobic glycolysis. Cell. (2013) 153:1239–51. 10.1016/j.cell.2013.05.016 23746840 PMC3804311

[39] Shi LZ Wang R Huang G Vogel P Neale G Green DR. HIF1alpha-dependent glycolytic pathway orchestrates a metabolic checkpoint for the differentiation of TH17 and Treg cells. J Exp Med. (2011) 208:1367–76. 10.1084/jem.20110278 21708926 PMC3135370

[40] De Bock K Georgiadou M Schoors S Kuchnio A Wong BW Cantelmo AR. Role of PFKFB3-driven glycolysis in vessel sprouting. Cell. (2013) 154:651–63. 10.1016/j.cell.2013.06.037 23911327

[41] Wang R Dillon CP Shi LZ Milasta S Carter R Finkelstein D. The transcription factor Myc controls metabolic reprogramming upon T lymphocyte activation. Immunity. (2011) 35:871–82. 10.1016/j.immuni.2011.09.021 22195744 PMC3248798

[42] Macintyre AN Gerriets VA Nichols AG Michalek RD Rudolph MC Deoliveira D. The glucose transporter Glut1 is selectively essential for CD4 T cell activation and effector function. Cell Metab. (2014) 20:61–72. 10.1016/j.cmet.2014.05.004 24930970 PMC4079750

[43] Palsson-McDermott EM Curtis AM Goel G Lauterbach MA Sheedy FJ Gleeson LE Pyruvate kinase M2 regulates Hif-1alpha activity and IL-1beta induction and is a critical determinant of the warburg effect in LPS-activated macrophages. Cell Metab. (2015) 21:65–80. 10.1016/j.cmet.2014.12.005 25565206 PMC5198835

[44] Kong Q Li N Cheng H Zhang X Cao X Qi T. HSPA12A Is a novel player in nonalcoholic steatohepatitis via promoting nuclear PKM2-mediated M1 macrophage polarization. Diabetes. (2019) 68:361–76. 10.2337/db18-0035 30455376

[45] Thomas SJ Snowden JA Zeidler MP Danson SJ. The role of JAK/STAT signalling in the pathogenesis, prognosis and treatment of solid tumours. Br J Cancer. (2015) 113:365–71. 10.1038/bjc.2015.233 26151455 PMC4522639

[46] Gao X Wang H Yang JJ Liu X Liu ZR. Pyruvate kinase M2 regulates gene transcription by acting as a protein kinase. Mol Cell. (2012) 45:598–609. 10.1016/j.molcel.2012.01.001 22306293 PMC3299833

[47] Wang B Liu S Fan B Xu X Chen Y Lu R. PKM2 is involved in neuropathic pain by regulating ERK and STAT3 activation in rat spinal cord. J Headache Pain. (2018) 19:7. 10.1186/s10194-018-0836-4 29349661 PMC5773456

[48] Yang P Li Z Li H Lu Y Wu H Li Z. Pyruvate kinase M2 accelerates pro-inflammatory cytokine secretion and cell proliferation induced by lipopolysaccharide in colorectal cancer. Cell Signal. (2015) 27:1525–32. 10.1016/j.cellsig.2015.02.032 25778902

[49] Skommer J Wlodkowic D Deptala A. Larger than life: mitochondria and the Bcl-2 family. Leukemia Res. (2007) 31:277–86. 10.1016/j.leukres.2006.06.027 16911824

[50] Liang J Cao R Wang X Zhang Y Wang P Gao H. Mitochondrial PKM2 regulates oxidative stress-induced apoptosis by stabilizing Bcl2. Cell Res. (2017) 27:329–51. 10.1038/cr.2016.159 28035139 PMC5339831

[51] Chen J Xie J Jiang Z Wang B Wang Y Hu X. Shikonin and its analogs inhibit cancer cell glycolysis by targeting tumor pyruvate kinase-M2. Oncogene. (2011) 30:4297–306. 10.1038/onc.2011.137 21516121

[52] Lee SY Kwok SK Son HJ Ryu JG Kim EK Oh HJ. IL-17-mediated Bcl-2 expression regulates survival of fibroblast-like synoviocytes in rheumatoid arthritis through STAT3 activation. Arthritis Res Ther. (2013) 15:R31. 10.1186/ar4179 23421940 PMC3672783

[53] Yang J Zhao S Yang X Zhang H Zheng P Wu H. Inhibition of B-cell apoptosis is mediated through increased expression of Bcl-2 in patients with rheumatoid arthritis. Int J Rheum Dis. (2016) 19:134–40. 10.1111/1756-185X.12706 26176566

[54] Singh D Arora R Kaur P Singh B Mannan R Arora S. Overexpression of hypoxia-inducible factor and metabolic pathways: possible targets of cancer. Cell Biosci. (2017) 7:62. 10.1186/s13578-017-0190-2 29158891 PMC5683220

[55] Azoitei N Becher A Steinestel K Rouhi A Diepold K Genze F. PKM2 promotes tumor angiogenesis by regulating HIF-1alpha through NF-kappaB activation. Mol Cancer. (2016) 15:3. 10.1186/s12943-015-0490-2 26739387 PMC4704385

[56] Lee YA Choi HM Lee SH Hong SJ Yang HI Yoo MC. Hypoxia differentially affects IL-1beta-stimulated MMP-1 and MMP-13 expression of fibroblast-like synoviocytes in an HIF-1alpha-dependent manner. Rheumatology. (2012) 51:443–50. 10.1093/rheumatology/ker327 22123992

[57] Kastenhuber ER Lowe SW. Putting p53 in context. Cell. (2017) 170:1062–78. 10.1016/j.cell.2017.08.028 28886379 PMC5743327

[58] Xia L Wang XR Wang XL Liu SH Ding XW Chen GQ. A novel role for pyruvate kinase M2 as a corepressor for P53 during the DNA damage response in human tumor cells. J Biol Chem. (2016) 291:26138–50. 10.1074/jbc.M116.737056 27810895 PMC5207082

[59] Wu HL Yang P Hu WL Wang YY Lu YX Zhang LC. Overexpression of PKM2 promotes mitochondrial fusion through attenuated p53 stability. Oncotarget. (2016) 7:78069–82. 10.18632/oncotarget.12942 27801666 PMC5363644

[60] Ao R Guan L Wang Y Wang JN. Effects of PKM2 gene silencing on the proliferation and apoptosis of colorectal cancer LS-147T and SW620 cells. Cell Physiol Biochem. (2017) 42:1769–78. 10.1159/000479456 28746922

[61] Seemayer CA Kuchen S Neidhart M Kuenzler P Rihoskova V Neumann E. p53 in rheumatoid arthritis synovial fibroblasts at sites of invasion. Ann Rheum Dis. (2003) 62:1139–44. 10.1136/ard.2003.007401 14644850 PMC1754413

[62] Simelyte E Rosengren S Boyle DL Corr M Green DR Firestein GS. Regulation of arthritis by p53: critical role of adaptive immunity. Arthritis Rheum. (2005) 52:1876–84. 10.1002/art.21099 15934085

[63] Roskoski R Jr. ERK1/2 MAP kinases: structure, function, and regulation. Pharmacol Res. (2012) 66:105–43. 10.1016/j.phrs.2012.04.005 22569528

[64] Keller KE Doctor ZM Dwyer ZW Lee YS. SAICAR induces protein kinase activity of PKM2 that is necessary for sustained proliferative signaling of cancer cells. Mol Cell. (2014) 53:700–9. 10.1016/j.molcel.2014.02.015 24606918 PMC4000728

[65] Feng J Ma T Ge Z Lin J Ding W Chen H. PKM2 gene regulates the behavior of pancreatic cancer cells via mitogen-activated protein kinase pathways. Mol Med Rep. (2015) 11:2111–7. 10.3892/mmr.2014.2990 25411978

[66] Schett G Tohidast-Akrad M Smolen JS Schmid BJ Steiner CW Bitzan P. Activation, differential localization, and regulation of the stress-activated protein kinases, extracellular signal-regulated kinase, c-JUN N-terminalkinase, and p38 mitogen-activated protein kinase, in synovial tissue and cells in rheumatoid arthritis. Arthritis Rheum. (2000) 43:2501–12. 10.1002/1529-0131(200011)43:11<2501::AID-ANR18>3.0.CO;2-K 11083274

